# Telomerase Inhibitory Effects of Red Pigment Rubropunctatin and Statin Monacolin L Isolated from Red Yeast Rice

**DOI:** 10.3390/genes8050129

**Published:** 2017-04-26

**Authors:** Baojun Xu, Qijun Wang, Changkeun Sung

**Affiliations:** 1 Food Science and Technology Program, Beijing Normal University-Hong Kong Baptist University United International College, Zhuhai 519085, China; 2 College of Food and Bioengineering, South China University of Technology, Guangzhou 510640, China; fewangqj@scut.edu.cn; 3 Department of Food Science and Technology, Chungnam National University, Taejon 305-764, Korea; kchsung@cnu.ac.kr

**Keywords:** red yeast rice, pigments, anticancer, apoptosis, telomerase

## Abstract

In addition to the cholesterol-lowering activity of red yeast rice (RYR), its anticancer activities have been frequently reported. However, the mechanism of action of the anticancer activity of RYR is not yet fully understood. The objective of the current study was to elucidate anticancer compositions and anticancer mechanism of actions of RYR. The isolated compounds from RYR were subjected to anti-proliferation assay, apoptosis assay via flow cytometry, and telomerase inhibitory assay via telomeric repeat amplification protocol-PCR (TRAP-PCR) assay, and Western blotting assay in an in vitro cell culture system. The results showed that a statin, monacolin L, and a red pigment, rubropunctatin, from RYR exhibited very strong cancer cell proliferation inhibitory effects; the rubropunctatin was comparable with anticancer drug *cis*-platinum, taxol, and 10-hydroxy-camptothecin (HCPT) in their IC_50_ values. Monacolin L and rubropunctatin exerted their anticancer activity via telomerase inhibitory effects. Monacolin L and rubropunctatin presented the similar telomerase inhibitory effects as the anticancer drug *cis*-platinum, while the anticancer drug HCPT presented a weak telomerase inhibitory effect in the TRAP-PCR assay. Meanwhile, rubropunctatin and *cis*-platinum did not present strong apoptosis induction activity as the momacolin L and HCPT did. These results indicate that the RYR may exert anticancer effects through the telomerase inhibitory effect of rubropunctatin and the apoptosis-induction effect of monacolin L.

## 1. Introduction

Red yeast rice (RYR), produced by fermenting *Monascus* species on steamed rice, is one paradigm of traditional Chinese medicinal food consumed in China. Clinical observations clearly showed that functional red yeast rice can lower blood-lipid levels in humans [1], and this function was partly due to the presence of cholesterol synthetase inhibitors. As cholesterol synthetase inhibitors, over 10 statin compounds were found in the metabolites of several fungal cultures. In the past two decades, some new pigments and metabolites have been isolated and characterized from red yeast rice [2,3,4,5]. Beyond cholesterol-lowering activities, antibiotic [6], immunosuppressive [7], and hypertension-alleviating activities [8] of red yeast rice have been found. Continuous discovery of new bioactive activities of *Monascus* metabolites have attracted more researchers to study *Monascus*.

In recent years, there have been emerging interests in statin drugs (analogues of monacolins) used as anticancer agents based on preclinical evidence of their anti-proliferative, pro-apoptosis, anti-invasive, and radio-sensitizing properties [9,10,11,12]. In addition, *Monascus* pigment dose-dependently reduced the incidence of skin tumor formation [13]. However, there are no reports on the mechanisms of action of anticancer activity of red yeast rice, except for one report in which the apoptosis and autophagic cell death of human prostate cancer cells induced by pigment monascuspiloin was reported [14]. Based on these considerations, red yeast rice, or its chemical compositions, are the hopeful anticancer products. In order to illustrate its anticancer compositions and mechanisms of actions, several monacolins and pigments were isolated from red yeast rice. The anticancer activities were further investigated by evaluating their anti-proliferation activity, apoptosis inducing activity, and telomerase inhibitory activity.

## 2. Materials and Methods

### 2.1. Chemicals and Materials

Trifluoroacetic acid (TFA), propidium iodide (PI), 3-(4, 5-dimethylthiazolyl-2)-2, 5-diphenyletrazolium bromide (MTT), *cis*-platinum, taxol, all trans-retinoic acid (ATRA), and 10 hydroxyl-camptothecin (HCPT) were purchased from Sigma-Aldrich Chemical Co. (St. Louis, MO, USA). Cell culture medium RPMI 1640 and fetal bovine serum (FBS) were purchased from GIBCO (Carlsbad, CA, USA). Penicillin-streptomycin and trypsin-versene mixture were purchased from BioWhittaker (Walkersville, MD, USA). Sterilized cell culture materials were purchased from Beckton Dickinson Labware (Franklin Labkes, NJ, USA).

### 2.2. Monascus Strain and Solid State Culture of Monascus

The solid-state culture of *Monascus ruber* on steamed rice was carried out in plastic mushroom bottles at 32 °C for 15 days. The solid medium consisted of 100 g rice, 14 g soybean powder, 2 g sucrose, and 1 g yeast extract according to our previous publication [15].

### 2.3. Isolation of Compounds from Red Yeast Rice

The monacolins and red pigments were isolated from red yeast rice (the solid culture of *Monascus ruber*) by column chromatographic methods. The isolation procedures have been reported in our latest publication [16].

### 2.4. Structural Identification of Isolated Compounds

**Figure 1:**
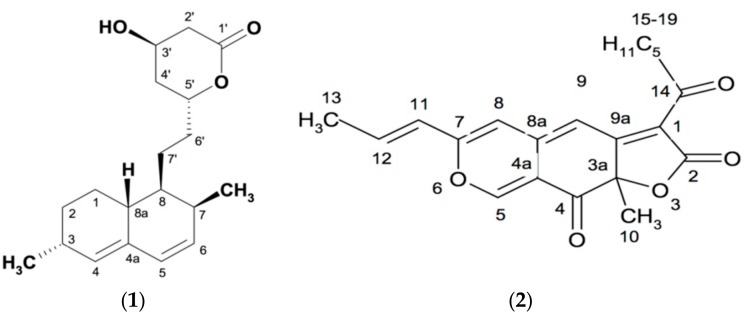
Chemical structures of monacolin L (**1**) and rubropunctatin (**2**).

The isolated compounds were identified as monacolin L and rubropunctatin, which were reported in our latest publication [16]. The chemical structural of two compounds are presented in Figure 1.

### 2.5. Cell Lines and Cell Culture

The human acute promyeloid leukemic cell line K-562, human ovary adenocarcinoma cell line SK-OV-3, and human stomach adenocarcinoma cell line SNU-1 were provided from the Korea Cell Line Bank (Seoul, Korea) and maintained in RPMI 1640 medium containing phenol red, supplemented with 10% FBS and with penicillin-streptomycin (100 U/mL) in 5% CO_2_, 95% air, at 37 °C.

### 2.6. Preparation of Test Sample Solutions

For making 1 mg/mL of stock solutions of compounds, 50 μL of DMSO were added into the Eppendorf tubes contained 1 mg isolated compounds, compound solutions were ultrasonicated for 20 min, followed by vortexing for 1 min, then 950 μL of PBS solution was added into tubes to reach a 1 mL volume. The stock solutions were stored at 4 °C for use. For preliminary cytotoxic screening on fast-growth cell line SNU-1, according to the evaluation criteria of NCI (IC_50_ value of a compound at less than 10 μg/mL was considered as a potential anticancer agent), 100 μg/mL of working solutions (the final concentration was 10 μg/mL in the MTT assay) were made by diluting 1 mg/mL of stock solution. For plotting cell viability curves and measuring IC_50_ values, the samples in which cell viability was less than 50% in the preliminary screening were selected out (IC_50_ ≤ 10 μg/mL), and gradient concentration solutions were further made by diluting 1 mL of stock solution with PBS buffer.

### 2.7. Cancer Cell Proliferation Inhibitory Assay

The anti-proliferation determination was performed using MTT assay. Briefly, cells were seeded into 96-wells culture plates at a seeding density of 1 × 10^4^ cells per well (SNU-1), 1.5 × 10^4^ cells per well (SK-OV-3) in 180 μL RPMI 1640 medium. The cells were cultured in an atmosphere of 95% air and 5% carbon dioxide at 37 °C and 90% humidity for 24 h before sample treatment. Subsequently, cells were exposed to the isolated compounds and positive anticancer drug control for 48 h. After the sample treatment, 20 μL MTT (5 mg/mL) was added to cultures for 4 h. Suspension cells (SNU-1) were centrifuged at 1760 rpm for 10 min, then the supernatant was removed. The formation of yellow formazan (a product of the reduction of tetrazolium by viable cells) was dissolved in 150 μL DMSO, and the mixture was gently shaken for 10–15 min under dark conditions. The absorbance of the DMSO solution at 540 nm was measured by an ELISA microplate reader (Molecular devices Emax, Sunnyvale, CA, USA). Softmax Pro 4.6 software was used for data processing. Cytotoxicity was evaluated by IC_50_ values and each assay was done in triplicate. Cell viability curves against the control were created by SigmaPlot 7.0 software. Morphological observation of cell viability was done by fluorescence microscopy.

### 2.8. Telomeric Repeat Amplification Protocol-PCR (TRAP-PCR) Assay

Telomerase activity was detected by TRAP assay described previously [17] and our recent publication [18]. Prior to TRAP-PCR assay, the protein content of K562 cell lysate was measured and adjusted to a suitable concentration to obtain a reliable TRAP-PCR assay. Protein estimation was performed by Bradford assay [19]. Two primers were synthesized by Bioneer Co. (Taejon, Korea) according to the previous published sequences [20]. TS: 5′-AATCCGTCGAGCAGAGT T-3′, CX: 5′-CCCTTACCCTTACCCTTACCCTAA-3′. The detailed TRAP-PCR procedures were reported in our previous publication [18]. After TRAP-PCR, 20 μL of the PCR products were loaded on a 12.5% non-denaturing polyacrylamide gel (Protean^®^ II xi Vertical Electrophoresis Cells, Bio-Rad, Hercules, CA, USA). Gel electrophoresis was performed at a constant voltage of 300 volts for 2 h. The gel was developed after silver staining. After gel staining, the gel was photographed by an Olympus digital camera and scanned by a Canon scanner. Ladder bands were analyzed with a Bio-Rad fluorescent multi-image system (Bio-Rad Laboratories, Hercules, CA, USA), and telomerase inhibitory activity was evaluated by comparing the light level of the detected ladders with the negative control.

### 2.9. Evaluation of Taq DNA Polymerase Inhibitory Activity

A conventional PCR reaction by adding previous telomerase positive fractions was carried out to evaluate the effects of isolated compounds on Taq DNA polymerase activity. The template DNA used in the general PCR reaction was from the genomic DNA of Chinese cabbage var. Chiifu, and the primer pairs were L: CTGCAGATAGAGAAGACAGACAAG and R: TCAGATCATCGGA TAGAGAAGACA. These materials were provided by the Molecular Breeding Laboratory of Chungnam National University (Daejeon, Korea).

### 2.10. Apoptosis Assay by Flow Cytometry

Flow cytometric quantification of apoptosis cells within the PI stained population was measured by FACS analysis. The cells with a higher percentage of M1-gated DNA content as compared to control one represents apoptotic cells.

### 2.11. Total Protein Assay

One milliliter (1 × 10^4^ cell/well) of SK-OV-3 cells was inoculated into 24-well tissue culture plates, respectively. After 48 h of culturing, isolated compounds (10 μg/mL) and anticancer-positive drugs (10 μg/mL) were added into the cultures. After another 48 h of culturing, suspension cells were transferred into Eppendorf tubes, while monolayer cells were digested by trypsin-EDTA, then transferred into Eppendorf tubes, and cells were harvested by centrifugation at 1000 rpm for 5 min. Cell pellets were washed by PBS twice by suspension and centrifugation. The washed cell pellets were stocked at −70 °C for use. Bradford protein assay [19] was used for the total protein assays. Briefly, 0.5 mL of lysis solution (0.3 M NaOH) was added into Eppendorf tubes containing cell pellets, and were incubated at 100 °C for 30 min. One-hundred microliters of cell lysis solution was mixed with 1.0 mL of Bradford reagent (10% Coomassie Brilliant Blue G-250 (*w*/*v*), 4.75% ethanol (*v*/*v*), 10% phosphoric acid (*w*/*v*)), after 10 min, absorbance of the mixtures of protein and Bradford reagent were measured at 595 nm on a UV-VIS spectrometer. The gradient concentration of BSA was used to make the standard curve for the calculation of the protein concentration.

### 2.12. Western Blot Analysis for Gene Expression of Human Telomerase Reverse Transcriptase (hTERT)

K562 cells with a density of 5 × 10^4^/mL were inoculated into six-well culture plates, after 24 h of culturing, cells were treated with or without compounds (10 μg/mL) for 1–2 days, and harvested by centrifugation at 500× *g* for 10 min. Cells were washed with ice-cold PBS, then stocked in −70 °C for use. The cell pellets were suspended in RIPA lysis buffer, and then placed on ice for 30 min. After centrifugation at 12,000× *g* for 15 min, cell lysate supernatants were collected. Protein concentration of cell lysate was determined by previous Bradford assay using BSA as a standard. After cell lysate was boiled for 5 min, 5–25 μL of cell lysate (30 μg of protein) was loaded into each well of 12% SDS-PAGE along with pre-stained protein molecular weight standard (MBI). After electrophoresis, protein bands were transferred to nitrocellulose membranes at a constant current of 250 mA for four hours in transfer buffer (25 mM Tris, 190 mM glycine, 20% MeOH). The blot was removed from the transfer apparatus and immediately placed into blocking buffer (5% non-fat dry milk, 10 mM Tris pH 7.5).

After incubating the blot overnight at 4 °C, blocking buffer was decanted from the blot, primary antibody solution (1:500 dilution) added and incubation continued with agitation for at 37 °C 1 h. The primary antibody solution was then decanted and rinsed with wash buffer. For secondary antibody incubation, the horse-radish peroxides-conjugate anti-mouse or rabbit IgG antibody was diluted (1:500) in wash buffer containing 5% non-fat dry milk and incubated with agitation for 30 min at 37 °C. The secondary antibody solution was then decanted and rinsed with wash buffer; the wash buffer was decanted and the blot placed in a plastic chamber containing chemiluminescent working solution. The membranes were then exposed to Kodak X-Omat AR films. Antibody to hTERT was used to detect the corresponding total protein levels.

### 2.13. Statistical Analysis

Data were expressed as means ± standard deviation (Std). Statistical analysis was performed using 2005 SAS (Version 9.1, SAS institute Inc., Cary, NC, USA). Duncan’s multiple range tests were used to determine the differences between group means. Significant levels were defined as probabilities of 0.05 or less. The inhibitory concentration 50% (IC_50_) was calculated from the concentration effect regression line. In each case, an appropriate range of 4–5 concentrations was used.

## 3. Results and Discussion

### 3.1. Telomerase Inhibitory Activities of Red Yeast Rice and Isolated Compounds

**Figure 2:**
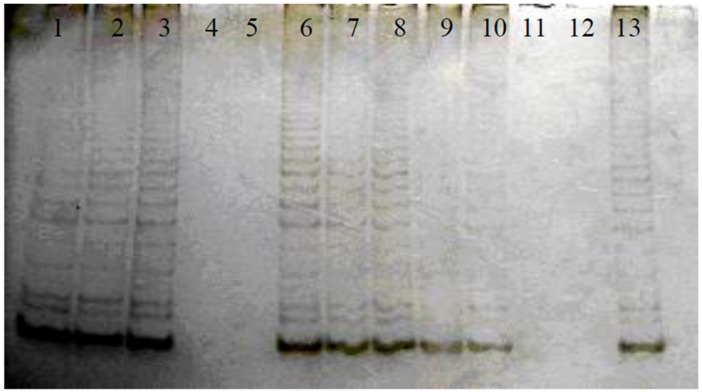
The effect of isolated compounds on telomerase activity. Lane 1: telomerase positive control cell(K-562); Lane 2: 0.5 μL of DMSO; Lane 3: negative control (lysis buffer substituted test samples); Lane 4: telomerase heat inactive cell lysate; Lane 5: anti-cancer drug *cis*-platinum; Lane 6: anti-cancer drug taxol; Lane 7: anti-cancer drug HCPT; Lane 8: ATRA; Lane 9: ethyl acetate extract of red yeast rice; Lane 10: monacolin K; Lane 11: monacolin L: Lane 12: rubropunctatin; Lane 13: positive control cell (K-562).

The standard TRAP assay utilizes a single tube reaction comprised of two steps: primer extension, followed by PCR amplification of the extension products. PCR reaction products were run on a native PAGE and stained with silver nitrate. The detection procedures of telomerase inhibitory activity have been reported in a previous publication of ours [18]. Therefore, the TRAP-PCR system was also applied for testing of telomerase inhibitory effects of red yeast rice, the isolated compounds from red yeast rice, and the anticancer positive drugs. As shown in Figure 2, the anti-cancer drug *cis*-platinum, monacolin L, and rubropunctatin presented positive telomerase inhibitory effects, while other anti-cancer drugs did not exhibit telomerase inhibitory effects. To avoid the potential false positive telomerase inhibitory results caused by the inhibition of Tag polymerase, the regular PCR were run by adding the isolated compounds and anticancer drugs. The compounds that did not inhibit the regular PCR were verified to be positive telomerase inhibitors. The results of TRAP-PCR and regular PCR were listed in Table 1. The regular PCR further confirmed the telomerase inhibitory effects of monacolin L, rubropunctatin, and *cis*-platinum.

### 3.2. Anti-Proliferation and Apoptosis Effects of Monascus Metabolites against Cancer Cells

**Figure 3:**
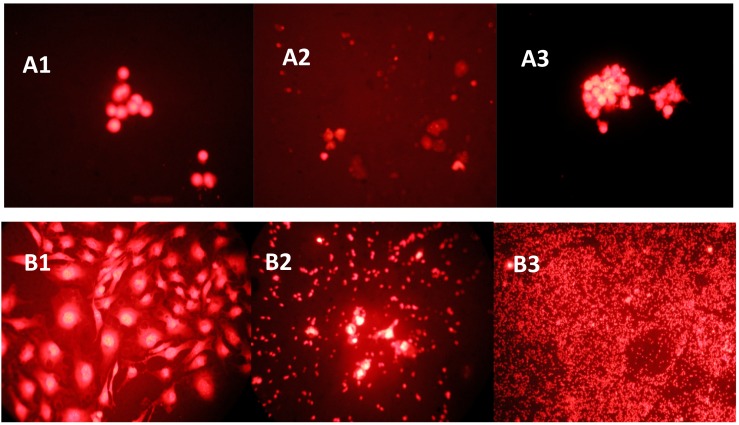
Morphological changes of cancer cells SNU-1 (**A**) and SK-OV-3 (**B**) after 48 h of exposure to the compounds (10 µg/mL). PI stained cells (A and B) were observed under a fluorescence microscope equipped with a red filter set at 400× magnification. **A1**,**B1**: control cells; **A2**,**B2**: cells exposed to rubropunctatin; **A3**,**B3**: cells exposed to positive anticancer agent *cis*-platinum and HCPT, respectively.

**Figure 4:**
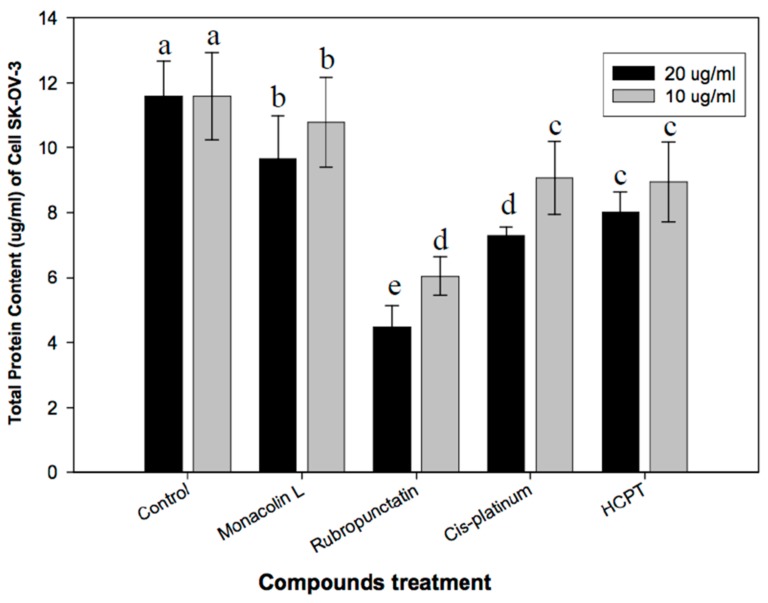
Protein content of cancer cell line SK-OV-3 treated by compounds. The same letters above the graphic bars within the same concentration group indicate no significant (*p* < 0.05) difference between different treatments.

**Figure 5:**
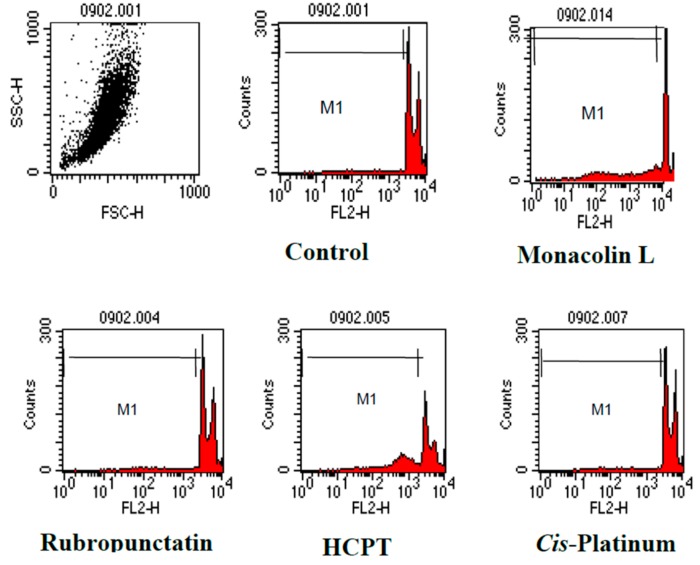
Flow cytometry histogram of cancer cell line K562. After 24 h exposure to the compounds (10 μg/mL), cells were harvested and stained with PI. DNA relative content was analyzed by flow cytometry. *X*-axis: DNA contents; *Y*-axis: number of cell.

The preliminary study on the anti-cancer effects of *Monascus* metabolites have been reported in our latest study [16]. The current study further investigated the anti-proliferation effects and its mechanisms of action. The morphological changes of cancer cells SNU-1 and SK-OV-3 upon exposure to rubropunctatin and anti-cancer drug *cis*-platinum were elucidated in Figure 3. The control cells showed normal living cells stained green (Figure 3A1,B1). Rubropunctatin-treated cells showed condensed nuclei, membrane blebbing, and apoptotic bodies (Figure 3A2,B2). The total protein contents of SK-OV-3 upon exposure to isolated compounds and anticancer drugs are elucidated in Figure 4. The anticancer drugs and rubropunctatin presented significant lower protein levels as compared to the control, meanwhile showing the dose-dependent inhibitory effect. The dose-dependent inhibitory effects of monacolin L, rubropunctatin, and anticancer drugs against cancer cell proliferation were presented in Table 2. Flow cytometry analysis (Figure 5) was applied to test cancer cell proliferation inhibitory mechanisms, monacolin L and anticancer drug HCPT presented apoptosis induction effects, while rubropunctatin and anticancer drug *cis*-platinum did not present apoptosis induction effects on K562 cells, which presented similar DNA relative contents in the M1 phase of cells as that of the control cells.

### 3.3. Expression of Human Telomerase Reverse Transcriptase (hTERT)

**Figure 6:**
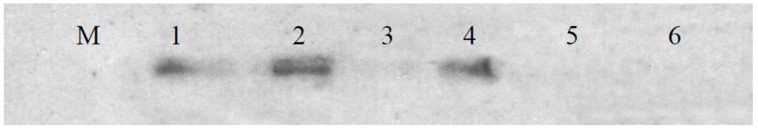
Expression of human telomerase reverse transcriptase (hTERT). K562 cells were exposed to 10 μg/mL of compound for 24 h. Expression of telomerase-related protein hTERT was performed by Western blot assay. The blot membranes were exposed on Kodak film. M: protein marker; Lane 1: K562 positive cells; Lane 2: anticancer agent HCPT; Lane 3: *cis*-platinum; Lane 4: taxol; Lane 5: monacolin L; Lane 6: rubropunctatin.

After finding telomerase inhibitory activity, anticancer activity, and apoptosis-inducing activity of the compounds, the mechanisms of telomerase inhibition were further investigated by measuring the expression of telomerase-related protein hTERT. The effects of the isolated active compounds on the expression of hTERT were investigated by Western blot analysis, and the Western blot results (Figure 6) showed that anticancer positive drug *cis*-platinum, and compounds monacolin L and rubropunctatin, completely suppressed the expression of protein hTERT, while anticancer agents HCPT and taxol did not affect the expression of hTERT. These results indicated that telomerase inhibition caused by monacolin L and rubropunctatin might be attributed to downregulation of telomerase-related protein hTERT.

In an in vitro cell study, Hong et al. [11] reported that other components in RYR, including other monacolins, pigments, or the combined matrix effects of multiple constituents may affect intracellular signaling pathways differently than purified crystallized lovastatin in colon cancer cells. The effect of mandolin rich-RYR was similar to that of lovastatin, while the effect of pigment rich-RYR was similar to that of the whole RYR extract in proliferation, apoptosis, and mRNA levels of HMGCR and sterol response element binding protein-2. These results suggest that matrix effects of RYR beyond lovastatin alone may be active in inhibiting colon cancer growth. Hong et al. further investigated the anticancer effects through a mouse model and found that cancer cell inhibition by RYR was greater than that observed with lovastatin at the dose found in RYR, showing that other compounds in RYR contributed to the anti-proliferative effect [21]. Epidemiological studies show that individuals taking statins have a reduced risk of colon cancer. These studies indicate that the health promotion effects of red yeast rice is not limited to cholesterol lowering effects, and red yeast rice has also presented anticancer effects in in vitro and in vivo studies. The statins in red yeast rice partially contribute to its anticancer activity; the other components, like pigments, also exert anticancer effects.

In our current study, activity-guided isolation yielded statins and red pigment (rubropunctatin) from red yeast rice, and an in vitro cell study indicate that monacolin L and rubropunctatin presented very strong anticancer effects against cancer cells. Our study further verified a previous hypothesis that some other components may also exert anticancer effects of red yeast rice. In fact, we found that red pigment rubropunctatin presented even stronger anticancer effects than anticancer drugs taxol and *cis*-platinum against ovary cancer cells. We further explored the anticancer mechanism through an apoptosis assay and telomerase inhibitory assay, finding that red pigment rubropunctatin exerts its anticancer effects through telomerase inhibitory effects by downregulating gene expression of telomerase-related protein hTERT.

It has been known that telomerase is expressed in the majority of human tumors; it has been used as a molecular marker for cancer diagnosis and therapeutic strategies. hTERT has been identified as a putative catalytic subunit of human telomerase [22]. Reconstitution experiments, both in vitro and in vivo, also strongly suggest that hTERT is the major determinant of human telomerase activity [23,24]. Over-expression of hTERT in cancer cells is thought to contribute to tumor development and angiogenesis [25]. Therefore, downregulation of hTERT might be one of the strategies for cancer treatment.

The current study suggests that red yeast rice and its red pigment component, rubropunctatin, deserve to be further studied on their anticancer effects and mechanisms of action. As a telomerase inhibitor, rubropunctatin may exert anticancer effects through suppressing gene expression of telomerase-related protein hTERT. More in-depth studies should be carried out to further develop it into an anticancer drug candidate.

## Figures and Tables

**Table:** 

	Control	Monacolin L	Rubropunctatin	HCPT	*cis*-Platinum
All-gated %	100	100	100	100	100
M1-gated %	4.79	32.97	6.65	39.72	5.66

**Table 1:** Telomerase inhibitory activity of compounds.

Sources	Compounds	TRAP-PCRinhibitory	Taq Polymerase Inhibitory Activity	Telomerase Inhibitory Activity
Anticancer drugs	Taxol	−	−	−
	*cis*-Platinum	+	−	+
	ATRA	−	−	−
	HCPT	W	−	W
Isolated compounds	Monacolin K	W	−	W
	Monacolin L	+	−	+
	Rubropunctatin	+	−	+

+: positive result; −: negative result; W: weak positive results. K-562 cells were used for study.

**Table 2:** Anticancer activity of isolated compounds against cancer cell lines SNU-1 and SK-OV-3.

Compounds	Cell Viability (%) of SNU-1 *							IC_50_ (µg/mL) **
Concentration	40 (µg/mL)	20 (µg/mL)	10 (µg/mL)	5 (µg/mL)	2.5 (µg/mL)	1.25 (µg/mL)	0 (µg/mL)	
Monacolin L	---	37.5 ± 3.4 e	48.9 ± 4.5 d	57.0 ± 6.2 c	58.5 ± 4.3 c	66.3 ± 5.9 b	100 ± 8.9 a	9.8
Rubropunctatin	10.4 ± 0.8	23.5 ± 2.5 e	55.6 ± 4.1 d	69.4 ± 6.4 c	83.7 ± 2.6 b	87.8 ± 1.9 b	100 ± 2.8 a	13.3
*cis*-Platinum	15.8 ± 3.9	26.5 ± 2.2 f	38.4 ± 3.3 e	53.1 ± 10.6 d	69.6 ± 6.6 c	78.0 ± 6.5 b	100 ± 3.3 a	8.1
HCPT	---	23.5 ± 8.8 d	25.2 ± 5.9 cd	29.3 ± 5.0 c	32.6 ± 8.4 bc	35.5 ± 0.6 b	100 ± 7.7 a	2.3
	Cell Viability (%) of SK-OV-3 *							
Monacolin L	---	68.6 ± 2.4 e	73.7 ± 1.9 d	84.3 ± 1.3 c	87.0± 1.3 bc	91.9 ± 6.2 b	100 ± 7.5 a	28.9
Rubropunctatin	---	25.4 ± 2.8 f	44.1 ± 1.3 e	64.3 ± 0.9 d	82.1 ± 3.5 c	92.8 ± 3.2 b	100 ± 3.1 a	8.2
Taxol	24.5 ± 1.3 g	34.4 ± 2.5 f	53.3 ± 3.7 e	67.9 ± 2.2 d	74.8 ± 0.9 c	85.0 ± 2.5 b	100 ± 4.2 a	11.4
*cis*-Platinum	18.3 ± 3.4 g	31.1 ± 1.3 f	53.3 ± 1.2 e	67.8 ± 1.0 d	79.6 ± 0.3 c	87.0 ± 0.7 b	100 ± 2.9 a	11.2

* Cell viability data were expressed as means ± std. from three parallel level treatments; ** IC_50_ was calculated from a third-degree polynomial fit by using Curve Expert 1 software. Values marked by the same letter within the same row are not significantly (*p* < 0.05) different.
